# Chaperone regulation of biomolecular condensates

**DOI:** 10.3389/frbis.2024.1342506

**Published:** 2024-05-06

**Authors:** Jared A. M. Bard, D. Allan Drummond

**Affiliations:** 1 Department of Biology, Texas A&M University, College Station, TX, United States,; 2 Department of Biochemistry & Molecular Biology, The University of Chicago, Chicago, IL, United States,; 3 Department of Medicine, Section of Genetic Medicine, The University of Chicago, Chicago, IL, United States

**Keywords:** cell stress, adaptation, chaperones, biomolecular condensate, stress granules, proteostasis

## Abstract

Biomolecular condensation allows for the dynamic organization of molecules in time and space. Condensate formation is regulated through many mechanisms including the action of molecular chaperones. While molecular chaperones have long been viewed through the lens of their roles in protein folding, misfolding, and quality control, their ability to manipulate protein-protein interactions is increasingly recognized to play a major role in the precise control of condensate biology. In this review we highlight recent studies investigating the roles of canonical and non-canonical chaperones in regulating condensate formation, material state, and dispersal. We discuss the broadening of longstanding conceptions of chaperone functions to include condensate regulation, and the discovery of previously unappreciated chaperone activities in well-known proteins. We close by considering the biological activities being uncovered during the ongoing upheaval at the boundary between chaperone biology and biomolecular condensation.

## Introduction

The regulation of reactions in cells requires the dynamic organization of macromolecules. In addition to being segregated by membrane-bound compartments, numerous processes in cells are organized into biomolecular condensates—membraneless, non-stoichiometric ensembles of macromolecules (Banani et al., 2017; Sabari et al., 2020; Lyon et al., 2021; Glauninger et al., 2022). Some of these condensates, such as the nucleolus, are constitutive features of cells which help organize basic housekeeping functions of the cell (Lafontaine et al., 2021). Others, such as stress granules, form transiently in response to an external signal (Glauninger et al., 2022).

Remarkable progress has been made in understanding the biophysical forces underlying condensation in cells and even reconstituting *in vitro* many condensates, both simple and complex. These studies have shown that the condensation behavior of many proteins is encoded directly in their amino acid sequences, even including the ability to modulate condensation based on environmental signals—often conceived of as stresses—such as heat, pH, and desiccation (Riback et al., 2017; Yoo et al., 2019; Iserman et al., 2020; Dorone et al., 2021). Early studies which helped jumpstart the ongoing revolution in condensate biology routinely showed not just that proteins, along with other biological molecules, formed condensates upon a change in conditions—concentration, ionic strength, pH, temperature, and so on—but also that condensation was spontaneously reversible when conditions returned to the prior state (Molliex et al., 2015; Nott et al., 2015). The loss of spontaneously reversible interactions was highlighted as a key step in the transition to pathological states, such as amyloid (Molliex et al., 2015).

Over time, the accumulation of results in multiple systems has modulated this picture considerably. Spontaneous reversal of nonpathological condensates gave way to regulated dispersal by posttranslational modification or the action of molecular chaperones (Yoo et al., 2022). The latter, one aspect of the chaperone-mediated regulation of biomolecular condensates which is the subject of this review, remains relatively new, and may be surprising, given entrenched models describing the nature of chaperone action and of chaperone substrates. Chaperones have long been conceived of as protein quality control systems, acting to combat toxic protein misfolding (proteotoxic stress) and restore protein homeostasis (proteostasis) when it is disrupted (Morimoto, 2008; Vabulas et al., 2010; Zhou et al., 2014; Ungelenk et al., 2016; Hua et al., 2022). It is abundantly clear that chaperones possess these activities, and express them when confronted with foreign misfolded aggregation-prone substrates. However, the endogenous substrates of chaperones remain remarkably ill-defined.

## Chaperones and condensates: beyond protein quality control

The present review is intended to promote reconsideration of some of these ideas in light of biomolecular condensation. In the simplest terms, the model in which proteins form toxic aggregates that can be cleaned up by chaperones must be expanded—and in some circumstances replaced—by a model in which proteins form adaptive condensates which are regulated at every step by chaperones.

A recent study serves to illustrate how the proteotoxicity and protein quality control perspective has become paradigmatic, in the sense of being an implicit frame which profoundly shapes interpretations, and yet is potentially misleading. In work focusing on the sequestrase activity of Btn2 (reviewed extensively here), ethanol-treated cells show similar insoluble protein levels to cells heat-shocked at 42°C, leading to the conclusion that “severe ethanol stress … causes the denaturation and aggregation of proteins in yeast cells” (Kato et al., 2019). One can see the paradigm at the edges of this framing. Yet it is increasingly clear that many stress-triggered condensates are adaptive, evolved, and unlike denatured aggregates. Specifically, 42°C heat shock has not been shown to cause either denaturation or proteotoxic aggregation of mature proteins; instead, dozens of proteins form reversible condensates which variously retain enzymatic activity, promote growth and translation during stress, form by phase separation driven by limited conformational changes which are conserved across species rather than denaturation and aggregation, and so on (Wallace et al., 2015; Riback et al., 2017; Iserman et al., 2020; Keyport Kik et al., 2024; Chen et al., 2024). To the subject of this review, chaperones interactions with thermally induced misfolded aggregates turn out to differ markedly from their interactions with thermally triggered condensates (Yoo et al., 2022).

In light of these developments, we suggest the reader keep an open mind. Where prior work has implied aggregation is toxic under conditions in which endogenous substrates are largely or completely unknown, and where key results have relied on the use of foreign aggregating reporters, small-molecule inhibitors, overexpression, or deletions, the door to an alternative adaptive condensation interpretation should remain open.

We also extend this open mind to the term biomolecular condensate itself, which we here use in the most broad sense, referring to any membrane-less cluster which concentrates specific macromolecules. This includes structures such as amyloids, in which high-affinity specific interactions link molecules together into a chain, or effectively a one-dimensional condensate. While amyloids are traditionally associated with toxic aggregation, there are now a plethora of examples of functional, adaptive, and reversible amyloids (Boke et al., 2016; Chuang et al., 2018; Woodruff et al., 2018; Cereghetti et al., 2021). That said, we will still attempt to distinguish between aggregates (inert, non-adaptive and misfolded assemblies of proteins) and condensates whose clustering we generally assume to be an evolved or at least adaptive trait.

With that in mind, here we will focus on regulation of condensation, broadly conceived, by molecular chaperones. The concept of a molecular chaperone has evolved and expanded over time, and we adopt an inclusive view. Chaperones were originally described as “proteins whose function is to ensure that the folding of certain other polypeptide chains and their assembly into oligomeric structures occur correctly” (Ellis, 1987). Although this initial definition also included a provision that chaperones not form part of the final assembly, subsequent work has relaxed this requirement to include chaperones, including small heat-shock proteins, which remain associated with larger-scale structures, potentially to alter their physical characteristics, and perhaps as a consequence of directing their formation. Indeed, the recognition that heat-shock proteins—literally, at the outset, proteins produced in response to heat shock—were rife with what soon came to be called molecular chaperones has led to use of these terms somewhat interchangeably and imprecisely.

The recent recognition that some intracellular aggregates are in fact biomolecular condensates, even in the case of heat shock itself, further justifies the stance we adopt here: chaperone activities include not just the prevention of oligomeric assemblies—even today synonymous with “chaperone activity”—but also promotion, modulation, and dispersal of oligomeric assemblies, of which condensates are an increasingly important example. This stance leaves out related activities, such as the posttranslational modification of molecules to regulate their condensation, while preserving the spirit of the chaperone concept. We will also leave out nucleic acid chaperones, such as RNA helicases, which play an important role in regulating biomolecular condensates containing both nucleic acids and proteins by regulating RNA-RNA (or DNA-DNA) and nucleic acid-protein interactions (Ripin and Parker, 2022). In Table 1, we have included a list of chaperones discussed in this review and the condensates that they regulate.

We begin with a brief overview of the biophysics of condensate formation, highlighting the thermodynamic and kinetic forces which enable the complex dynamics and structures of condensates. Chaperones are able to finely tune these forces to regulate all stages of the condensate lifecycle, and we organize our review accordingly, highlighting the ways in which chaperones promote and direct the formation of condensates; regulate condensate size, dynamics, and internal structure; and promote condensate dispersal. We close with discussion and review of the functional consequences of condensate regulation by chaperones, highlighting open challenges in understanding the biology of these still-enigmatic processes.

## Integrating chaperones into a conceptual framework of condensation

While biomolecular condensate formation is often compared to simple systems such as the phase separation of oil and water, the underlying molecular forces governing complex biological polymers are much more complex. We will not attempt a comprehensive review of the biophysics underlying condensates, instead referring readers to many excellent and detailed reviews on the subject (Brangwynne et al., 2015; Banani et al., 2017; Choi et al., 2020; Mittag and Pappu, 2022). As a very brief intro, biomolecular condensates generally require multivalent interactions in order to form a three dimensional network (Li et al., 2012; Brangwynne et al., 2015; Choi et al., 2020). One useful model for these multivalent interactions is that interacting “stickers” are connected by a flexible “spacer” (Choi et al., 2019). This model can be adapted to interaction surfaces as small as individual amino acids and as big as entire proteins. The propensity of a system to phase separate is determined by the relative strength of the interactions between the stickers, spacers and solvent. Stronger sticker-sticker interactions and weaker sticker-solvent interactions will promote condensation. The strength of these interactions is not just an intrinsic property of the molecule, but is dependent on a variety of environmental factors, including salt concentrations, temperature and pH.

**FIGURE 1:**
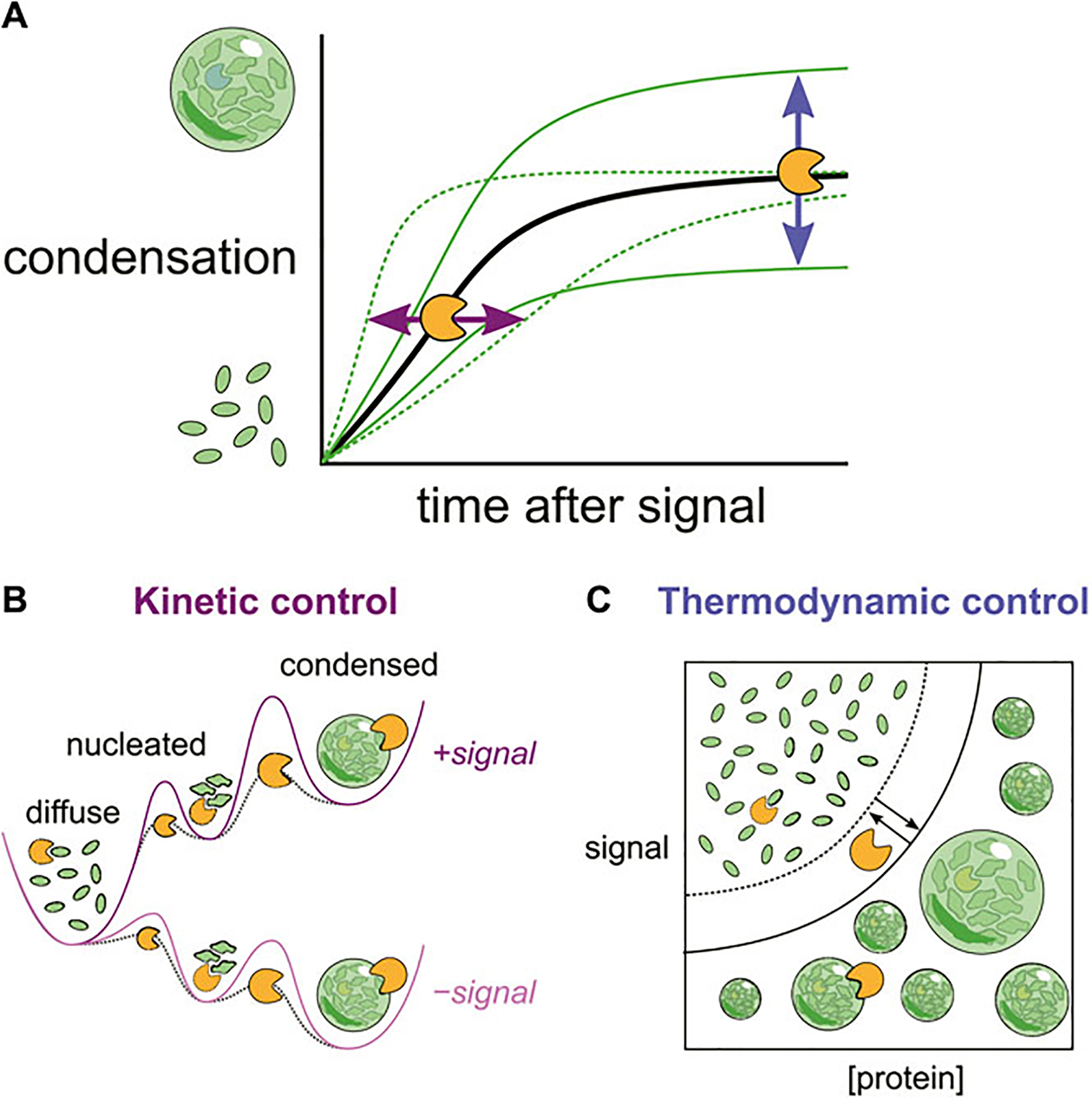
**(A)** Changes in the cellular environment can trigger biomolecular condensation. Chaperones can reshape both the **(B)** kinetics (rates) and **(C)** thermodynamics (levels) of condensation by interactions at multiple stages.

Importantly these interactions also set a critical concentration of the condensing molecules themselves. Condensation is only energetically favorable above this concentration. Because the critical concentrations reflect the current cellular environment, any changes to that environment can change the transition threshold, causing condensates to form or dissolve without changes in the concentration of the molecules. These cellular changes could include direct environmental signals such as heat or osmolarity changes, the appearance of a new species such as dsRNA from a virus or changes in the concentration of a secondary messenger such as cAMP(Riback et al., 2017; Jalihal et al., 2019; Iserman et al., 2020; Zhang et al., 2020; Dorone et al., 2021; Wen and Ma, 2022; Watson et al., 2023). Chaperones can also change this critical concentration by competing for those same intramolecular interactions that drive condensation. This is then thermodynamic control of condensates, in which chaperones change the underlying energy landscape to favor or disfavor condensation (Figure 1).

While phase transitions can happen quickly, they are not instantaneous. Even in an energetic landscape where condensation is favorable, there is a kinetic barrier to converting soluble molecules to condensates and *vice versa*. Here too chaperones play a key role. In fact, modulating barriers between intermediate states is the principle mechanism by which chaperones accomplish their canonical task of encouraging proteins to reach their most energetically favorable form (Hartl et al., 2011). By modulating the kinetic barrier between soluble and condensed states, chaperones can accelerate or inhibit condensate formation and dispersal, which in turn can have important functional implications for cellular growth and adaptation (Figure 1).

## Chaperones promote and direct biomolecular condensation

Alongside the well-known roles of chaperones in preventing aggregation, dispersing aggregates, and assisting with folding, a wide range of studies have uncovered roles for chaperones—primarily small heat-shock proteins and functional homologs—in promoting and directing localization of condensation. The overwhelming majority of work on such activities of chaperones, which are termed sequestrases, aggregases, or condensases in this context (Kedersha et al., 2016; Ho et al., 2019; Mogk et al., 2019; Weis, 2021; Shrivastava et al., 2022; Carter et al., 2023), has focused on formation of aggregates or deposits of misfolded, denatured proteins en route to degradation, with sequestration thought to serve a cytoprotective function (Shrivastava et al., 2022). As described in the Introduction, we adopt the perspective that adaptive condensation coexists with such quality control activities.

Our knowledge of sequestrase activity has come overwhelmingly from studies in budding yeast, where, after considerable efforts, multiple groups have converged on a common high-level understanding of the structures and players involved (Kaganovich et al., 2008; Malinovska et al., 2012; Miller et al., 2015). Yeast forms three major quality control structures apparent as foci by microscopy: a cytosolic quality control compartment called CytoQ, an intranuclear quality control compartment called INQ, and a perivacuolar insoluble protein deposit called IPOD (Kaganovich et al., 2008; Miller et al., 2015; Miller et al., 2015). Although these structures are commonly referred to as “compartments,” the nature of their boundaries remains unclear, apart from their lack of a membrane.

How precisely these proteostatic compartments form, and how the activity of sequestrase molecules promotes the appearance of microscopically visible structures, is not yet fully known. However, their appearance is regulated by two key chaperone proteins, the major sequestrases in budding yeast: CytoQ by the small heat-shock protein Hsp42, and INQ by the small heat-shock protein Btn2.

**FIGURE 2:**
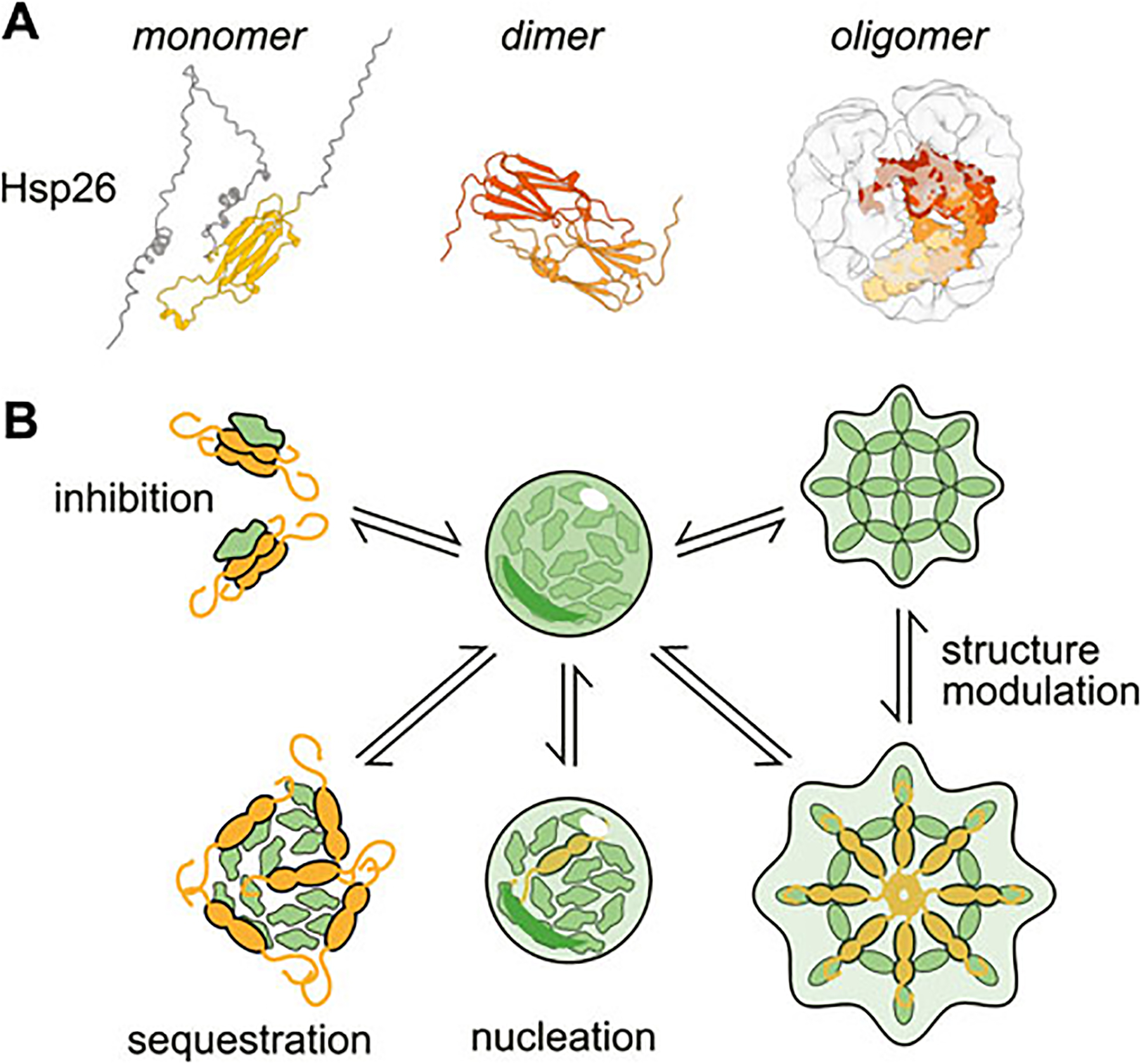
Structure and activities of a canonical small heat-shock protein (sHSP). **(A)** Structures of the small heat shock protein Hsp26 as a monomer (AlphaFold prediction (Jumper et al., 2021; Varadi et al., 2022)), dimer (only structured α-crystallin domain shown, ColabFold prediction (Mirdita et al., 2022) and oligomer (cryo-EM structure with modeled monomers of Hsp26 (Mühlhofer et al., 2021)). **(B)** Examples of the ways small heat shock proteins can modulate condensation.

The small heat shock protein (sHsp) class of chaperones (Figure 2) consist of a structured α-crystallin domain (ACD) flanked by unstructured regions on the 5’ (NTD) and 3’ (CTD) ends (Haslbeck and Vierling, 2015). Interactions between these disordered regions and the ACD enable sHSP oligomerization, forming a range of species from dimers to 40-mers (Clouser et al., 2019; Mühlhofer et al., 2021). The exact mechanism of substrate binding varies between clients, but can involve binding sites in either the unstructured termini or the structured domain (Reinle et al., 2022). Unlike many other molecular chaperones, sHSPs do not hydrolyze ATP. Instead it is thought that they form a shell around misfolded proteins, thus preventing the substrate from forming aggregates (Friedrich et al., 2004; Shi et al., 2013; Żwirowski et al., 2017; Johnston et al., 2021; Mühlhofer et al., 2021). While sHSPs can form large oligomers, it is actually thought that oligomerization can inhibit sHSP activity by sequestering substrate-binding sites (Franzmann et al., 2008; Peschek et al., 2013; Jovcevski et al., 2015; Freilich et al., 2018).

Like many small heat-shock proteins, Btn2 forms large oligomers in the absence of stress (Ho et al., 2019). Although the precise structure of these oligomers remains unclear, Btn2 may behave similarly to other sHsps, with partial oligomer breakdown creating interaction sites for substrates (Haslbeck et al., 2019). In certain models of complete oligomers, such as polyhedral structures, the intrinsic multivalence of such incomplete oligomers could provide a scaffold for recruiting multiple substrates, driving sequestration, perhaps through nucleation. In addition to its role in INQ formation, Btn2 sequesters prion-associated Ure2p amyloid filaments in mother cells, such that after cell division, some daughter cells are cured of the prion (Kryndushkin et al., 2008; Son and Wickner, 2022).

An important clue to Btn2’s structure and function comes from its paralog in *Saccharomyces cerevisiae*, Cur1, which shares the Btn2 ACD but lacks Btn2’s long, disordered C-terminal domain (CTD) (Miller et al., 2015). Deletion of Btn2 disrupts INQ and CytoQ formation, and overexpression of Btn2 promotes formation of both structures, whereas deletion of Cur1 has a minimal effect whether deleted or overexpressed (Malinovska et al., 2012). Together, these results suggest that it is Btn2’s disordered CTD that is crucial for its sequestrase activity. A similar result holds for Hsp42, whose N-terminal prion-like disordered domain is required for CytoQ formation and aggregase activity *in vitro* (Grousl et al., 2018).

Hsp42 is the primary sequestrase active in the yeast cytoplasm, driving CytoQ formation—earlier called peripheral aggregates or Q-bodies—in response to proteotoxic conditions (Specht et al., 2011; Malinovska et al., 2012; Grousl et al., 2018). Like other sHSPs, it forms oligomers, with the most-populated wild-type state consistent with a 10-mer (Grousl et al., 2018). Unlike other sHSPs, these oligomers remain intact even upon heat stress (Haslbeck et al., 2004). Hsp42 and Btn2 both help to suppress the aggregation and toxicity of stress-induced aggregating proteins (Carter et al., 2023), although their endogenous targets outside of prion proteins remain obscure. We now turn our attention toward the roles of both proteins in regulating condensation and the focus-forming compartments CytoQ and INQ.

What is the relationship between condensation promoted by sequestrase action and the appearance of cellular foci? The naive expectation—that they are two expressions of the same underlying phenomenon—may not hold, as it does not hold in other related systems. Cycloheximide (CHX) inhibits CytoQ formation (Grousl et al., 2018), interpreted to mean that newly synthesized polypeptides make up the bulk of CytoQ substrates (Boczek and Alberti, 2018). However, disruption by CHX is a hallmark of other structures, notably P bodies (Sheth and Parker, 2003; Cougot et al., 2004; Kshirsagar and Parker, 2004) and stress granules (Mazroui et al., 2002; Mollet et al., 2008; Zhou et al., 2014; Wallace et al., 2015), with a different interpretation: that these structures depend on ribosome-free RNA for their formation. A key result is that translation inhibition with cycloheximide or emetine, which lock ribosomes on mRNAs, prevents granule formation, whereas inhibition with puromycin, which releases ribosomes from mRNAs, promotes granule formation (Glauninger et al., 2022). To our knowledge, a comparable experiment for either CytoQ or INQ has not been performed. It may be useful to consider the possibility that these compartments are the result of elaborate cell-biological processes, and that the *in vitro* aggregation experiments probe only early stages of subassembly formation, as has been found for stress granules (Wallace et al., 2015; Glauninger et al., 2022).

Questions regarding the seeding of condensed structures naturally lead to the core concept of nucleation. In the above examples, mRNA may serve as a nucleus (formally) or a scaffold (less formally) on which other biomolecules condense. sHsps, in their role as sequestrases, may use nucleation to spatially direct condensation; a simple cellular strategy would be to direct sequestrases to the compartments in which they should promote condensation. Indeed, deletion of Btn2, compromising INQ, can be rescued by expressing Hsp42-NLS, a construct which drives cytosolic Hsp42 into the nucleus using a nuclear localization sequence (NLS) (Ho et al., 2019).

Nucleation is likely to be the principal kinetic barrier to formation of many intracellular condensates (Martin et al., 2021; Shimobayashi et al., 2021). Condensation without a nucleation barrier can occur under conditions where nuclei form spontaneously and, as a result, condensation proceeds immediately everywhere in the solution—spatial control of where condensates form is lost. Thus, biological systems in which spatial control is beneficial will tend to have nucleation barriers. And similar to many familiar examples, from snowflakes nucleated by dust grains to steam bubbles nucleated by boiling chips, nucleation sites can be provided by auxiliary and often lower-abundance components present with the condensing species. This separation of roles creates a key niche for chaperones to act as sequestrases.

Condensates can be directed to specific locations not just by nucleation (directing formation), but also by tethering. Here, the subcellular tethering of age-associated aggregates by the Hsp40 chaperone Ydj1 provides an illuminating example. Aging yeast cells gradually accumulate protein aggregates consistent with CytoQ bodies (e.g., marked by Hsp104) (Saarikangas and Barral, 2015). Rejuvenation of new daughter cells depends on asymmetric retention of these aggregates in the aging mother cell. Retention depends on diffusion barriers (Clay et al., 2014) and also on Ydj1, with a twist: although abundant in the cytosol, Ydj1 has a subpopulation which is farnesylated and tethered to the cytoplasmic side of the ER membrane. Disruption of this tethered Ydj1 population compromises asymmetric large-aggregate retention, perhaps by client-mode binding of smaller aggregate precursors (Saarikangas et al., 2017).

Less well-studied relative to quality-control structures are foci—putative condensates—formed by the protein Std1 which form under glucose-replete conditions and disassemble when cells switch to respiration (Simpson-Lavy et al., 2017). Here, remarkably, the disaggregase Hsp104 acts as a sequestrase: Δhsp104 cells cannot form Std1 foci, inhibiting Hsp104 with low levels of guanadinium hydrochloride reduces focus formation, and Hsp104 is recruited to, perhaps integral to, these foci (Simpson-Lavy et al., 2017). Hsp40 (Sis1) and Hsp70 (Ssa1) chaperones also contribute to focus formation and associated phenotypes, as do Btn2 and Cur1, suggesting the involvement of multiple aspects of the sequestrase and disaggregase systems in regulating these structures. That Std1 foci are present under what in most other studies is a non-stress, wild type condition, and that Std1 appears to be an endogenous substrate rather than a primary regulatory factor for these foci, makes this system particularly tantalizing. Further work, particularly at the biochemical level, is needed to clarify how apparently opposing functions from proteins best known for dispersal activities contribute to condensate assembly in this case.

## Chaperones regulate the material state of condensates

In addition to their role in promoting the formation of condensates, chaperones also regulate the internal structure, dynamics, and other *in situ* features of condensates, which we broadly refer to as the material state. Most commonly, a single feature of condensate material state, dynamics, is measured using techniques such as fluorescence recovery after photobleaching (FRAP) and single particle tracking (Alshareedah et al., 2021; Wang et al., 2022). While such measurements do report on the dynamics of molecules in the condensates, they provide only limited insight into the underlying molecular structure of the condensate. For instance, solid-like materials can either be gels, which are characterized by cross-linked networks of polymers swollen with solvent, or as glasses, which are disordered materials with a high barrier to reorganization (Jawerth et al., 2020; Mittag and Pappu, 2022). Furthermore, even simple two-component systems can form complex structures with multiple distinct phases coexisting (Garaizar et al., 2022). Cellular biological condensate structures can contain hundreds of different components, and can have correspondingly complex internal structures and organizations. Furthermore, they may be composites of multiple types of condensates. Much research remains to be done linking these material states with the adaptive role of condensates in cells, but we will highlight some examples below in which chaperones have been shown to play an important role in modulating condensate structure and we will discuss the possible functional implications of this regulation.

A number of recent results have shown that chaperones can alter the material state of condensates by ‘fluidizing’ the structure. That is, chaperones increase the rate at which the condensate can internally reorganize. In multiple instances this fluidization appears to be important for keeping condensates in a material state that is easily dispersible. This is analogous to the long known function of small heat shock proteins in maintaining protein aggregates in a more easily re-solubilized state (Kampinga et al., 1994; Ehrnsperger et al., 1997; Lee et al., 1997; Cashikar et al., 2005; Ungelenk et al., 2016; Yoo et al., 2022).

For instance, molecular chaperones help maintain a liquid-like state for peri-nucleolar condensate-containing orphan ribosomal protein subunits (Ali et al., 2023). These condensates form in response to heat shock in both yeast and humans and they recruit molecular chaperones including the Hsp40 Sis1/DNAJB6. Hsp40s are an ATP-independent class of molecular chaperones that are thought to be primarily substrate recognizing adapters for the ATP-dependent Hsp70 chaperone (Kampinga et al., 2019). Ali et al. were able to study the condensates in a lysate system, where either adding a chemical inhibitor of Hsp70 or depleting ATP slows down the dynamics of the condensate. Depleting Sis1/DNAJB6 from the nucleus delays the dispersal of the ribosomal protein condensates during recovery from stress, matching a report that inhibition of Hsp70 delays resolubilization of ribosome biogenesis proteins after heat shock in human cells (Sui et al., 2022). Importantly this delayed dispersal affects the fitness of yeast, delaying the resumption of cell growth after stress (Ali et al., 2023).

A similar interplay between chaperones and condensates contributes to the resumption of growth for yeast spores after exiting dormancy (Plante et al., 2023). More than 100 proteins condense during spore dormancy and are then resolubilized upon the resumption of growth. Deletion of the small heat shock protein Hsp42 delays the dispersal of these condensates. While its mechanism of action is still unknown, Hsp42 may be acting to maintain proteins in a state that is more easily solubilized during recovery.

Condensates again regulate the material state of the selective autophagy receptor p62/SQSTM1, which forms condensates around damaged lysosomes in order to initiate their degradation via lysophagy (Gallagher and Holzbaur, 2023). Hsp27 is recruited into these condensates and when Hsp27 is depleted the condensates are less liquid-like (as measured by FRAP) and initiate lysophagy more slowly. This fits with results from a slightly different system showing that maintaining liquidity of condensates formed by autophagy cargo is important for efficient selective autophagy (Yamasaki et al., 2020).

Another chaperone-regulated condensate is the purinosome, a multienzyme condensate which forms upon changes in growth conditions which upregulate purine biosynthesis (An et al., 2008; French et al., 2013; Kyoung et al., 2015; Pedley et al., 2018; 2022). Two components of purinosomes, PPAT and FGAMS, directly interact with Hsp90 (Pedley et al., 2018). Furthermore, inhibiting Hsp70 or Hsp90, another ATP-dependent molecular chaperone, prevents the formation of intact purinosomes and instead triggers the formation of condensates containing FGAMS alone (French et al., 2013). These FGAMS condensates are more solid-like than normal purinosomes, as assayed by their reduced sphericity (Pedley et al., 2022). Because the FGAMS condensates do not contain the entire suite of enzymes necessary for purine biosynthesis, Hsp90 is likely playing an important role in maintaining functional purinosome condensates. However, as is the case with the ribosomal protein condensates above, the mechanism by which chaperones tune the condensate properties of the purinosome remains to be uncovered.

Chaperones have also been shown to play a key role in modulating the material state of multiple condensing proteins involved in neurodegenerative disease, including FUS and TDP-43. FUS and TDP-43 are normally nuclear proteins involved in RNA metabolism, but are found in cytoplasmic inclusions in degenerating neurons that are key hallmarks of the neurodegenerative diseases amyotrophic lateral sclerosis (ALS) and frontotemporal dementia (FTD) (Harrison and Shorter, 2017). Mutations in FUS which alter its phase-separating properties are linked to ALS and FTD, suggesting that the regulation of FUS condensate structure is important for maintaining healthy neurons (Murakami et al., 2015; Patel et al., 2015; Harrison and Shorter, 2017; Murray et al., 2017; Portz et al., 2021). While reconstituted FUS and TDP-43 form condensates that are initially liquid-like, they harden and develop into amyloid-like fibrils over time (Kato et al., 2012; Molliex et al., 2015; Patel et al., 2015; Gasset-Rosa et al., 2019). This tendency could be due to the high protein concentrations experienced in liquid condensates which promote oligomer formation. Due to their importance for neurodegenerative diseases, a number of studies have looked at chaperone involvement in preventing and dispersing aggregates and condensates of amyloid-prone RNA-binding proteins including FUS, TDP-43 and Ataxin-2 (Ciechanover and Kwon, 2017). We will not cover the large body of literature examining the role of chaperones in preventing and dispersing misfolded aggregates and amyloid fibers of these and other proteins (Wentink et al., 2019), but will rather focus on the interaction between chaperones and the more liquid-like condensates of these proteins.

A number of chaperones have been shown to help maintain this liquid-like state of FUS and TDP-43 and prevent amyloid fiber formation. For FUS, this includes ATP-independent chaperones like the Hsp40 chaperone DNAJB1 and the small heat shock proteins Hsp27 and HspB8 (Gu et al., 2020; Liu et al., 2020; Boczek et al., 2021; Li et al., 2022). The ATP-dependent Hsp70 chaperones HSPA1A and HSPA8 are also reported to prevent amyloid formation of FUS condensates (Li et al., 2022). Similarly, HspB1, another small heat shock protein, helps maintain TDP-43 condensates in a liquid-like state (Lu et al., 2022). In cells, depleting HspB1 delayed the dispersal of TDP-43 condensates, again emphasizing the potential role of material state in regulating the dispersal kinetics of condensates (Lu et al., 2022).

In general, the ATP-independent chaperones help to maintain liquid-like states by first partitioning into condensates and then interfering with the protein-protein interactions that drive oligomer formation and the transition to a more solid-like state. For instance, both Hsp27 and HspB8 use their unstructured N-terminal domains (NTDs) to interact with FUS condensates (Liu et al., 2020; Boczek et al., 2021). For Hsp27 this interaction is tuned by stress-induced phosphorylation in the NTD of Hsp27(Liu et al., 2020). While unphosphorylated Hsp27 completely prevents FUS condensation *in vitro*, a phosphomimetic variant of Hsp27 rather co-partitions with the FUS condensate. Meanwhile, HspB8 does not prevent FUS condensation, but does partition into them (Boczek et al., 2021). Removing the CTD largely prevented multimerization of Hsp27, but did not abolish chaperone activity, consistent with previous studies showing that oligomerization of Hsp27 actually inhibits chaperone activity (Franzmann et al., 2008; Peschek et al., 2013; Jovcevski et al., 2015; Freilich et al., 2018; Mühlhofer et al., 2021). Intriguingly, it was recently reported that purified Hsp40 proteins can form condensates themselves (Gu et al., 2020). In addition, the same disordered region of the protein that enables homotypic condensates also promotes co-condensation of Hsp40 with FUS, while a different domain is required to prevent FUS fibrilization. Similarly, while the NTDs of Hsp27 and HspB8 are crucial for controlling the interaction of the chaperone with the FUS condensate, it is actually the α-crystallin-like domain (ACD) which is required to modulate the material state of the condensate (Liu et al., 2020; Boczek et al., 2021). The ACD interacts with the RNA recognition motif (RRM) domain of FUS, which is not normally involved in FUS condensation and may prevent the local RRM unfolding which has been hypothesized to drive fibril formation (Boczek et al., 2021).

As regards TDP-43, HspB1 binds to a region which is thought to form a transient helix involved in phase separation, suggesting that it may be acting to regulate the material state by directly weakening the interactions involved in promoting condensation. This same region of TDP-43 is bound by a variety of chaperones *in vitro* including Hsp40, Hsp70 and Hsp90 type chaperones (Carrasco et al., 2023). HspB1 also binds to the RRM domain of TDP-43, which, as it is for FUS, appears to be important for amyloid fibril formation (Chen et al., 2019; French et al., 2019). This is also reminiscent of the condensation mechanism of poly(A)-binding protein (Pab1), in which interactions between locally unfolded RRM domains drives assembly (Chen et al., 2024). These results highlight that it is not just interactions between chaperones and disordered regions that regulate condensation, but that interactions with seemingly folded domains can also be critical for modulating condensation formation and structure.

While cytoplasmic TDP-43 condensates are enriched for HspB1, TDP-43 also forms nuclear condensates which are associated with Hsp70s rather than small HSPs(Udan-Johns et al., 2014; Gu et al., 2021; Yu et al., 2021; François-Moutal et al., 2022). Yu et al. found that RNA-binding deficient TDP-43 forms condensates with a remarkable internal structure, which they termed an “anisosome” (Yu et al., 2021). The TDP-43 anisosome contains a liquid-like core of Hsp70 and is surrounded by a shell of ordered (and hence anisotropic), but liquid-like TDP-43. This structure is dependent on the ATPase activity of the Hsp70 chaperone, as inhibiting the chaperone *in vivo* causes the structure to become a well-mixed liquid that ages over time to a more solid-like state (Gu et al., 2021). The anisome structure is not unique to TDP-43, as Zhu et al. recently identified a similar structure with a core of Hsp70 and a shell of the nuclear protein REV-ERBα (Zhu et al., 2023). REV-ERBα is involved in the repression of the circadian clock in mouse livers, and the anisosome condensates appear to be important for this repressive function. Further research is needed to identify other proteins which may form chaperone-associated anisomes, and to determine both the molecular mechanisms of how they form and what the functional consequences of this intricate structure is.

## Chaperones inhibit and dissolve condensates

The best-studied function of molecular chaperones is to prevent and disperse aggregates of misfolded proteins. Chaperones accomplish this task by disrupting unwanted protein-protein interactions and untangling networks of protein polymers (Hartl et al., 2011). These same functions make chaperones ideal regulators of biomolecular condensation, which similarly involve a multitude of protein-protein interactions and potentially interwoven networks of biological polymers. In fact, as discussed below, chaperones appear to be much more efficient at clearing biomolecular condensates than at dispersing aggregates of misfolded proteins (Yoo et al., 2022). While there are some constitutive condensates in cells, such as the nucleolus, many condensates form in response to an environmental signal. Once the signal has dissipated or the cell has regained homeostasis, the induced condensate will then be dissolved.

Stress granules are a key example of a signal-induced condensate which is known to be dispersed by molecular chaperones. Stress granules are assemblies of proteins and RNA that form in the cytosol in response to cellular stresses such as hypoxia, starvation, heat shock, oxidative stress and viral infection (Glauninger et al., 2022). They are often defined by the visible appearance of foci containing mRNA and key marker proteins such as G3BP1/2, poly(A)-binding protein (Pab1) and other translation initiation factors such as the eIF3 complex. However, their composition is complex and stress-dependent, making a precise definition of stress granules difficult. Reflecting this ambiguity, the function of stress granules is an active area of research with proposed functions ranging from regulating translation and RNA metabolism to serving as a platform for innate immune signaling (White and Lloyd, 2012; Reineke et al., 2015; Deater et al., 2022; Mateju and Chao, 2022). Recently they have even been shown to play a key role in maintaining the integrity of endolysosomal membranes (Bussi et al., 2023).

Stress granules are thought to assemble in stages, driven by the condensation of multiple different proteins with RNA. Many of these proteins, including G3BP1, PABP, and Ded1/DDX3, condense in a test tube in response to physiological triggers (e.g., heat shock or long ribosome-free RNA) (Riback et al., 2017; Kroschwald et al., 2018; Guillén-Boixet et al., 2020; Iserman et al., 2020; Yang et al., 2020). For instance, purified Pab1 forms condensates when exposed to heat shock in a test tube (Riback et al., 2017). The condensation of Pab1 is not driven by its intrinsically disordered regions, though they do play an important role in modulating the condensation behavior. Rather the assembly process involves interactions between locally unfolded regions of Pab1’s RRM domains (Chen et al., 2024). These condensates are initially liquid-like, but quickly mature into a more solid-like state which does not dissolve after the protein has been returned to room temperature. This is similar to the *in vitro* behavior of Ded1, another stress granule component which condenses in response to elevated temperature (Iserman et al., 2020).

**FIGURE 3:**
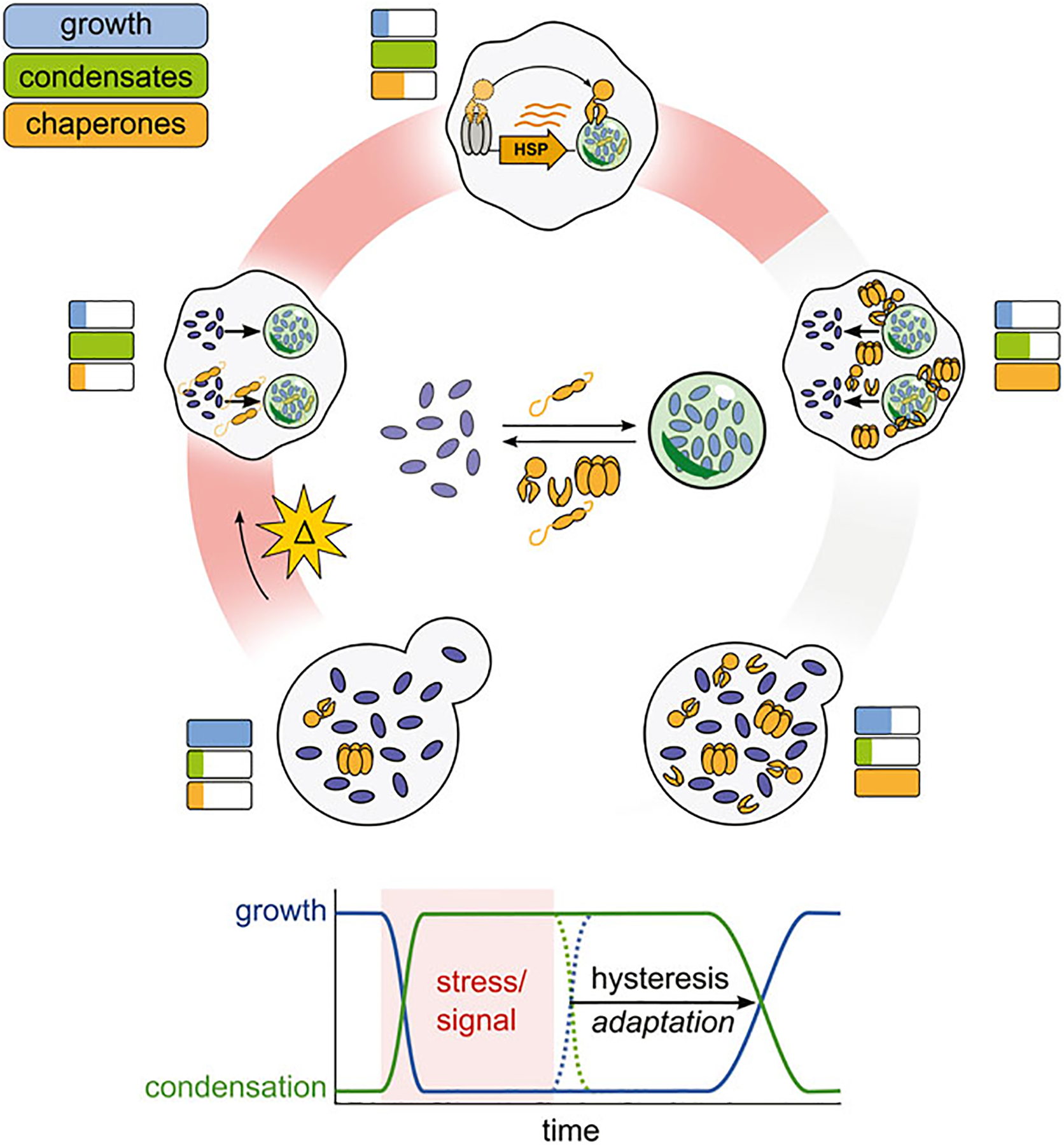
A proposed chaperone-enabled adaptive cycle of condensation. Stressful changes in cellular environments arrest cellular growth and trigger the formation of condensates, nucleated by small heat shock proteins. Stress-induced condensates then help to upregulate chaperone production, which begin to antagonize condensate formation. When stress ends, condensates do not spontaneously dissolve, but are dispersed by stress-induced chaperones, restoring growth. This lag (hysteresis) is adaptive, enabling cells to prepare for successive stresses and ensuring a successful response to stress before growth restarts.

Stress granules also do not immediately resolve upon the cessation of the stress. Thus both stress granules in cells and stress granule proteins *in vitro* demonstrate hysteresis, where the state of the system is influenced not only by its current conditions but also by past conditions. As we will discuss below, this property provides a useful regulatory handle by which chaperones can control the adaptation process of cells (Figure 3). Mechanistically, it could reflect a strong kinetic barrier to dissolution (Figure 1).

Stress granules are dissolved through a combination of at least two pathways: clearance and degradation by autophagy and resolubilization of the components by molecular chaperones (Buchan et al., 2013; Ganassi et al., 2016; Gwon et al., 2021). In yeast, condensation of proteins into stress-induced assemblies after a brief 42°C heat shock is completely reversible, suggesting that, at least for such short-term stresses, autophagy is not required for dissolution. Instead, dissolution is driven by the Hsp40/Hsp70/Hsp104/Hsp110 molecular chaperone pathway (Cherkasov et al., 2013; Kroschwald et al., 2015; Walters et al., 2015; Kroschwald et al., 2018; Yoo et al., 2022; To et al., 2023). In this pathway, the chaperones Hsp70, together with its co-chaperones Hsp40 and Hsp110, deliver substrates to the ring-shaped AAA+ unfoldase Hsp104, which uses energy from ATP hydrolysis to thread proteins through its central pore and resolubilize its substrates (Hartl et al., 2011).

Yoo *et al*. recently reconstituted this process *in vitro* using Pab1 condensates as a model substrate (Yoo et al., 2022). The full complement of chaperones from the pathway are required for maximal efficiency, but a combination of Hsp40, Hsp70 and either Hsp104 or Hsp110 is sufficient to reconstitute dispersal activity, with Hsp104-driven dispersal being significantly faster than that driven by Hsp110. While type I (Ydj1 in yeast) and type II (Sis1) Hsp40 proteins act synergistically in the disassembly of luciferase aggregates, only the type II Hsp40 is able to disperse Pab1 condensates (Nillegoda et al., 2015; Nillegoda et al., 2017). Another key point is that Hsp70, despite being able to catalytically hydrolyze ATP, needs to be present in super-stoichiometric abundance in order to disperse the condensates. This fits with other studies showing that multiple Hsp70s need to bind to a substrate in order to activate Hsp104 (Seyffer et al., 2012; Carroni et al., 2014). Similarly, even in the absence of Hsp104, multiple Hsp70s cluster together in order to disperse amyloid substrates (Wentink et al., 2020). In order to understand the regulation of stress granules and other condensates, more research is needed to identify the mechanisms enabling particular chaperones to disperse particular substrates.

A surprising finding was that dispersal of Pab1 condensates is orders of magnitude faster than dispersal of aggregates of luciferase, a commonly used model substrate, formed under similar conditions (Yoo et al., 2022). Computational modeling suggests that the difference in efficiency might be attributable to a higher dispersal rate of the substrate, and/or to the fact that, in contrast to luciferase, Pab1 remains monomeric after solubilization and does not reaggregate or reenter the condensate. Supporting contributions of the second mechanism, intrinsically disordered regions aid in refoldability of proteins, and condensing proteins in budding yeast are particularly adept at reforming their native structure after being unfolded (To et al., 2023). This suggests that proteins in condensates may have evolved properties such as fast refolding which allow them to be easily dispersed by molecular unfoldases like Hsp104.

While stress granule dispersal in budding yeast requires Hsp104, metazoa have lost this chaperone and yet are still able to disperse stress granules independently of autophagy (Erives and Fassler, 2015; Mateju et al., 2017; Wang et al., 2019; Gwon et al., 2021). In humans, this dispersal is enabled by VCP, a AAA+ ring-shaped unfoldase that is structurally quite similar to Hsp104 (Gwon et al., 2021). VCP is recruited to stress granules through its adaptor FAF2, which binds to ubiquitinated G3BP1. This is analogous to the recruitment of Hsp104 to stress granules by Hsp40/Hsp70 and suggests that VCP may have adopted some of the functions in regulating condensates performed by Hsp104 in fungi and other eukaryotes.

The Hsp70 system still plays an important role in regulating stress granules in humans, however, as it helps to maintain stress granules in a dispersible state (Ganassi et al., 2016; Mateju et al., 2017). Depletion of the Hsp70 HSPA1A, its co-chaperone BAG3, and the small heat shock protein HspB8 all lead to the accumulation of defective ribosomal products (prematurely terminated polypeptides) in stress granules, which in turn changes their material state and delays their dispersal (Ganassi et al., 2016). Similarly, overexpression of misfolded proteins, such as ALS-associated variants of the protein SOD1 leads to aberrant stress granules which are particularly dependent on chaperones for their dispersal (Mateju et al., 2017). Thus chaperones act across the life-cycle of stress granules to maintain their proper structural state, enabling them to be efficiently dispersed once the stress is relieved.

In addition to direct dispersal, chaperones can also control stress granules via indirectly modulating stress granule regulators. In particular, the active state of DYRK3, a kinase which regulates stress granule dissolution, is maintained by the ATP-dependent chaperone Hsp90 (Wippich et al., 2013; Mediani et al., 2021). Upon stress, DYRK3 is inactivated, disassociates from Hsp90 and is targeted to stress granules. During recovery, Hsp90 re-engages with DRYK3, reactivating it and further promoting stress granule dissolution. This system thus acts as a feed-forward system, sharpening the transitions between stress granule formation and dissolution. It also further ties chaperone activity to stress granules.

Stress granules are not the only condensate dispersed by the Hsp40/Hsp70 system. Another example of such condensates are those formed by RIα, a cAMP-dependent protein kinase (PKA) which aids in the regulation of cAMP signaling (Zhang et al., 2020). In turn, cAMP signaling regulates numerous pathways in cells. In liver cancer fibrolamellar carcinoma RIα is fused to the Hsp40 DNAJB1, which recruits Hsp70 and leads to the dispersal of RIα condensates. Similarly, direct targeting of Hsp70 to model condensates of RIα or EML4-Alk via *de novo* designed Hsp40 mimics also led to condensate dispersal (Zhang et al., 2023). This result opens up the exciting possibility of using chaperones as designer tools for modulating specific condensates in cells. Such tools would be similar to a number of variants of Hsp104 which have been specifically designed to disperse toxic aggregate associated with neurodegenerative diseases (Jackrel et al., 2014; Mack et al., 2023).

While the chaperones discussed above are general protein chaperones or unfoldases, a number of studies have highlighted the unexpected chaperone activities of other condensate binding proteins such as nuclear/cytoplasmic transport machinery (Guo et al., 2018; Hofweber et al., 2018; Qamar et al., 2018; Yoshizawa et al., 2018; Guo et al., 2019; Bourgeois et al., 2020; Hutten et al., 2020; Niaki et al., 2020; Baade et al., 2021; Khalil et al., 2022; Odeh et al., 2022; Rhine et al., 2022; Boeynaems et al., 2023; Thomas et al., 2023). Many of the RNA-binding proteins whose aberrant condensation is associated with neurodegenerative diseases, including FUS, TDP-43 and hnRNPA1, are trafficked into the nucleus via importins like Karyopherin-β2 (Kapβ2 or transportin 1) and KPNB1. Importins bind to proteins containing a nuclear localization sequence, and then use the energy from a concentration gradient of their regulator RAN to facilitate directional travel across the nuclear pore. Surprisingly, importin binding to FUS, TDP-43, hnRNPA1 and cold-inducible RNA-binding protein (CIRBP) inhibits their condensation and fibrilization both *in vivo* and *in vitro* (Guo et al., 2018; Hofweber et al., 2018; Qamar et al., 2018; Yoshizawa et al., 2018; Bourgeois et al., 2020; Hutten et al., 2020; Niaki et al., 2020; Baade et al., 2021; Khalil et al., 2022; Rhine et al., 2022). Even more impressive, despite having no ATP hydrolysis activity, Kapβ2 can dissolve preformed fibrils of FUS and hnRNPA1 *in vitro* (Guo et al., 2018; Fare et al., 2023). It accomplishes this dispersal by interacting with the substrate not just at the NLS but at multiple sites within the protein and thereby competing with the interactions that drive condensation and fibrilization (Guo et al., 2018; Yoshizawa et al., 2018; Fare et al., 2023). Similarly, the exportin CRM1 inhibits the formation of condensates of FG repeat-containing nucleoporins that appear when the nucleoporins are expressed at high levels (Milles et al., 2013; Thomas et al., 2023).

In a different example of unexpected chaperoning activity, the stress granule marker Pab1 (or PABPC in humans) also serves as a chaperone to prevent spontaneous condensation of ataxin-2 and to direct its localization to stress granules during cellular stress (Boeynaems et al., 2023). PABPC binds to a conserved short linear motif (SLiM) in ataxin-2, and is proposed to function by bringing ataxin-2 to RNA, which then competes with the multivalent interactions that otherwise lead to spontaneous condensation. In the context of stress, PABPC then acts as an emulsifier to allow ataxin-2 mixing with other stress granule components. In the absence of the SLiM, and thus PABPC binding, ataxin-2 does not mix with stress granules but rather forms condensates that dot the outside of stress granules. Together with the nuclear transport results, these findings force us to expand our definition of condensate chaperone beyond the traditional heat shock proteins.

## Biology enabled by chaperone-regulated condensation

The functions of chaperones in regulating condensates are, unsurprisingly, both as varied and as poorly understood as the functions of condensates themselves—determination of which is currently (and properly) considered a central aim of condensate biology.

We begin by noting and nodding to the standard protein quality control literature, extensively cited above, which has illuminated the many ways in which chaperones may act in the context of condensates to reduce the cellular impact of misfolded proteins. Prevention and dispersal of toxic condensates/aggregates, formation of quality control compartments such as the INQ and CytoQ, retention of similar bodies in aging cells, and alteration of the dispersability of aggregates by sHSPs represent the major activities attributable to chaperones and reviewed extensively above and elsewhere (Miller et al., 2015; Sontag et al., 2017; Haslbeck et al., 2019; Kumar et al., 2022). From the perspective of condensate biology, the key principles underlying the chaperone-regulated features of these systems are the sequestration of potentially toxic species into defined structures where their processing can be controlled and toxicity limited. A major open question is the degree to which these same structures and strategies apply to endogenous, adaptive condensing proteins—where, for example, sequestration of an activity which is deleterious in certain circumstances may follow the same logic as sequestration of toxicity.

Another relatively well-understood functional role of chaperones in regulating condensates is chaperone-mediated propagation of prions (Chakrabortee et al., 2016; Chakravarty et al., 2020; Itakura et al., 2020; Garcia et al., 2021; Saad and Jarosz, 2021). Prions are proteins which are able to propagate a particular conformational state, usually that of a multimeric assembly, across generations. These inherited states can have important functional consequences for cells, such as balancing a trade-off between proliferation and lifespan (Garcia et al., 2021). Many prions are deleterious, and chaperones form a key part of antiprion systems, in part by sHSP-mediated sequestration of potentially toxic prion assemblies to prevent their passage to younger cells (Kryndushkin et al., 2008; Son and Wickner, 2022). In other cases, chaperones break up prion assemblies which otherwise could not be transmitted, creating transmissible seeds which template future assembly growth (Shorter and Lindquist, 2004). The degree to which prions and condensates can and should be considered related phenomena remains somewhat hazy, but the understanding of condensation has been profoundly shaped by studies of prions, prion-like domains, sequestrases in a prion context, etc., enough to warrant substantial further attention.

Moving beyond quality control and toxicity, yet still in the realm of stress biology, two general functions of chaperones interacting with condensates have become clear in recent studies: regulating the timing of events following stress, and a crucial role in regulating responses to stress.

First, a common theme among stress-induced condensates is that their dissolution corresponds temporally to resumptions in the cellular processes which were suppressed during the stress. By regulating the timing of this dissolution, chaperones are playing a vital role in gatekeeping the return to normal cellular homeostasis. For instance, resumption of translation and the cell cycle after heat shock corresponds well with the dissolution of stress granules in yeast (Cherkasov et al., 2013; Kroschwald et al., 2018). It was also recently shown that in budding yeast availability of the chaperone Ydj1 regulates the cell-cycle (Moreno et al., 2019). Sequestration of Ydj1 into stress granules may thus directly delay resumption of the cell-cycle (Walters et al., 2015). Stress also triggers condensation of the ATP-producing enzyme Cdc19 into reversible amyloids which inhibit its function and must be disassembled before growth can restart (Cereghetti et al., 2021). Delaying the resolution of stress granules, on the other hand, delays the resumption of translation (Wippich et al., 2013; Maxwell et al., 2021). Similarly, delaying the resolution of orphan ribosomal protein condensates delays resumption of growth after heat shock (Ali et al., 2023). Another example comes from restarting growth in budding yeast spores, which corresponds with a large-scale resolubilization of proteins which had condensed during dormancy (Plante et al., 2023).

Second, chaperones appear to play a central role in transducing stress sensed by condensing proteins. Condensation and the more specific phenomenon of phase transitions represent an effective strategy to convert small changes in a relevant parameter (temperature, pH, etc.) into large-scale changes in intracellular organization (Yoo et al., 2019). However, to trigger downstream behavior, such sensory condensation must be read out, directly or indirectly. In the case of activation of Hsf1, the primary eukaryotic transcriptional regulator of the heat shock response, a mechanism for direct readout has already been described (Pincus, 2020). Under physiological growth conditions, Hsf1 is bound and repressed by the chaperone Hsp70(Zheng et al., 2016; Krakowiak et al., 2018). As new Hsp70 substrates emerge during stress, they titrate Hsp70 away from Hsf1, thus removing the inhibitory block and inducing chaperone induction. What are these substrates? One major source of new substrates for Hsp70 are nascent polypeptides which may misfold during cellular stress (Xu et al., 2016; Masser et al., 2019). However, even when translation is inhibited, stress leads to robust induction of the heat shock response (Triandafillou et al., 2020), suggesting that there is another source of Hsp70 substrates induced by stress. Indeed, poly(A)-binding protein (Pab1) both autonomously condenses *in vivo* and *in vitro* in response to temperature and pH changes (Riback et al., 2017), and when condensed is an endogenous substrate of the classic disaggregase system, including Hsp70 (Yoo et al., 2022). We hypothesize that many such stress-induced condensates, which are both abundant and interact strongly with chaperones during stress, also function as strong activators of Hsf1. Further supporting this adaptive role in regulating the stress response, we have recently shown that proteome-wide condensation in fungi is well-tuned to their evolutionary niches, with the temperature onset of condensation closely following the thermal niche of cryophilic, mesophilic and thermotolerant budding yeast species (Keyport Kik et al., 2024).

Temperature and pH trigger Pab1 condensation and, remarkably, similar structural rearrangements promoting condensation (Chen et al., 2024). Intracellular pH drops accompany multiple cellular stresses (Munder et al., 2016; Triandafillou et al., 2020), and have been proposed to be a second messenger for stress (Dechant et al., 2010). Generalized, these results suggest a relatively simple mechanism by which condensing proteins can respond to a diverse array of stresses, yet, via recruitment of chaperones acting as inhibitory factors on response proteins such as Hsf1, trigger convergent responses.

Given that chaperones appear to both be induced by stress-induced condensates and aid in their dispersal, we propose that chaperones and condensates have evolved as an adaptive monitoring system similar to negative feedback loops that govern many other signaling pathways (Figure 3) (Lake et al., 2016). To articulate the full picture, in this model stress triggers an arrest of growth and cellular processes such as translation, and also the induction of condensates, a process which itself is regulated by constitutive chaperones like small heat shock proteins (Rowley et al., 1993; Grousl et al., 2009). The condensates then aid in maintaining the repressed state by sequestering mRNA, translation initiation factors, and other proteins required for normal cellular growth (Riback et al., 2017; Bresson et al., 2020; Iserman et al., 2020). Condensates begin to recruit molecular chaperones away from Hsf1, relieving Hsf1 inhibition and triggering the induction of more molecular chaperones (Keyport Kik et al., 2024). This process continues until an equilibrium is established between condensate formation, condensate induction, and condensate dispersal. Autonomous stress-triggered condensation but facilitated dispersal dependent on limited chaperones produces hysteresis: condensates which form rapidly upon stress do not dissolve rapidly upon cessation of the triggering stress. Rather, condensates persist, generating a lag phase until sufficient chaperone activity exists to fully dissolve condensates. During this lag, the cell persists in a stress-specific state, with alterations in activities due to condensation then able to remodel the cell via selective transcription and translation to contend with or react appropriately to stress. Eventual condensate dissolution, in turn, corresponds with the resumption of the cell cycle and high-level translational activity (Cherkasov et al., 2013; Kroschwald et al., 2018; Moreno et al., 2019). This hysteretic system, in short, provides a simple mechanism by which the cell can generate specific responses and automatically detect a successful response, rather than merely the cessation of stress.

All of these functions, in their various states of illumination and mechanistic detail, provide merely a glimpse into the potential functions which may be executed by chaperones acting as regulators of condensation. We anticipate that the frame will continue to shift and expand to accommodate more adaptive mechanisms, more surprising reconsiderations of well-established chaperone activities, and a deeper understanding of condensate biology in the context of conserved regulatory machinery.

## Figures and Tables

**TABLE 1:** Chaperones discussed in the review along with the condensates that they regulate.

Chaperone class	Chaperone Name(s)	Promotes condensation of…	Modulates condensate structure of…	Prevents/disperses condensates of…
Hsp40	Sis1	Std1 (Simpson-Lavy et al., 2017)	orphan ribosomal proteins (Ali et al., 2023)	stress granules (Cherkasov et al., 2013; Kroschwald et al., 2015; 2018; Walters et al., 2015; Yoo et al., 2022; To et al., 2023)
Hsp40	DNAJB1		FUS (Gu et al., 2020)	RIα (Zhang et al., 2020)
Hsp70	Ssa1/2/3/4	Std1 (Simpson-Lavy et al., 2017)	purinosome (French et al., 2013; Pedley et al., 2018; 2022)	stress granules (Cherkasov et al., 2013; Kroschwald et al., 2015; 2018; Walters et al., 2015; Yoo et al., 2022; To et al., 2023)
Hsp70	HSPA1A/HSPA1/HSPA5/HSPA6/HSPA8		TDP-43 (Udan-Johns et al., 2014; Gu et al., 2021; Yu et al., 2021; François-Moutal et al., 2022); REV-ERBα(Zhu et al., 2023)	RIα (Zhang et al., 2020, 2023); EML4-Alk (Zhang et al., 2023)
Hsp90	Hsp90α/Hsp90β		purinosome (French et al., 2013; Pedley et al., 2018; 2022)	
AAA+	Hsp104	Std1 (Simpson-Lavy et al., 2017)		stress granules (Cherkasov et al., 2013; Kroschwald et al., 2015; 2018; Walters et al., 2015; Yoo et al., 2022; To et al., 2023)
AAA+	VCP			stress granules (Erives and Fassler, 2015; Mateju et al., 2017; Wang et al., 2019; Gwon et al., 2021)
sHSP	Btn2	Ure2 (Kryndushkin et al., 2008; Son and Wickner, 2022); INQ (Malinovska et al., 2012); CtyoQ (Malinovska et al., 2012; Simpson-Lavy et al., 2017)		
sHSP	Hsp42	CytoQ (Specht et al., 2011; Grousl et al., 2018)	sporulation-induced condensates (Plante et al., 2023)	
sHSP	Cur1	Std1 (Simpson-Lavy et al., 2017)		
sHSP	Hsp27		p62/SQSTM1 (Gallagher and Holzbaur, 2023); FUS (Liu et al., 2020)	
sHSP	HspB1		TDP-43 (Lu et al., 2022)	
sHSP	HspB8		FUS (Boczek et al., 2021)	
nuclear transport receptors	Kapβ2/KPNB1			FUS (Guo et al., 2018; Hofweber et al., 2018; Qamar et al., 2018; Yoshizawa et al., 2018; Bourgeois et al., 2020; Niaki et al., 2020; Baade et al., 2021; Rhine et al., 2022); TDP-43 (Guo et al., 2018; Hutten et al., 2020; Khalil et al., 2022); TAF15 (Guo et al., 2018); EWSR1 (Guo et al., 2018); hnRNPA1 (Guo et al., 2018); hnRNPA2 (Guo et al., 2018)
nuclear transport receptors	CRM1			nucleoporins (Thomas et al., 2023)
Other	PABPC		ataxin-2 (Boeynaems et al., 2023)	ataxin-2 (Boeynaems et al., 2023)
